# Supplementary material for: Population pharmacokinetic modeling of missed mycophenolate mofetil doses: Impact on exposure and exploration of mitigation strategies
Source: PLoS One. 2025 Aug 28;20(8):e0330854. doi: 10.1371/journal.pone.0330854 (PMC12393703; doi:10.1371/journal.pone.0330854)
Supplement: S1 File — (PDF) [file pone.0330854.s001.pdf]

````
```{r}
library(mrgsolve)
library(stats)
library(tidyverse)
library(truncnorm)
library(ggplot2)
library(conflicted)
library(PKNCA)
library(Pmetrics)
library(skimr)
library(openxlsx)
library(stats)
library(tidyr)
library(pander)
library(DT)
library(gridExtra)
library(cowplot)
library(dplyr)
library(knitr)
library(DT)
```
````

##Population Pharmacokinetics of Mycophenolic Acid Co-Administrated with Tacrolimus in Corticosteroid-Free Adult Kidney Transplant Patients, Yan Rong, 2019

````
```{r}
code <-
"
[SET] end=100, delta=0.1

[PARAM] @annotated
TVTlag : 0.162 : value lag time (h)
TVKA : 1.98 : Typical value of intercomp absorbance (1/h)
TVCL : 2.87 : Typical value of Clearance (L/h)
TVVC : 25 : Typical value of central volume (L)
TVQ : 36.7 : value of intercomp clearance (L/h)
TVVP : 607 : value of periph volume (L)
B1 : - 0.09 : covariate parameter estimate mycophenolic acid acyl- glucuronide
B2 : 0.68 : covariate parameter estimate


[PARAM] @annotated @covariates
AcMPAG : 0.54 : Mycophenolic acid glucuronide C0 (mg/L)
AUCMPAG : 588.8 : AUC Mycophenolic acid glucuronide (mg.h/L/g)
AUCMPA : 53 : AUC mycophenolic acid (mg.h/L/g)
````
````

```
[OMEGA] @annotated
ETATlag : 1.16 : IIV on Tlag
ETAKA : 0.98 : IIV on Ka absorbance
ETACL : 0.05 : IIV on apparent clearance
ETAVC : 0.03 : IVV on Central compartment volume of distribution
ETAQ  : 0.07 : IVV on apparent intercompartmental clearance
ETAVP : 1.16 : IVV on apparent peripheral compartment volume of distribution


[SIGMA]
0.0001
0.0001

[CMT] @annotated
GUT : estomac [ADM]
CENT  : Central compartment [OBS]
PERIPH : peripheral compartment



[MAIN]


double CL = TVCL*pow(AcMPAG,B1)*pow(AUCMPAG/AUCMPA,B2)*exp(ETACL);
double VC = TVVC * exp(ETAVC);
double KA = TVKA * exp(ETAKA);
double Q = TVQ * exp(ETAQ);
double VP = TVVP * exp(ETAVP);
ALAG_GUT = TVTlag * exp(ETATlag);


[ODE]
dxdt_GUT    = -KA*GUT;
dxdt_CENT   = -(CL+Q)*CENT/VC + Q*PERIPH/VP + KA*GUT;
dxdt_PERIPH = Q*CENT/VC - Q*PERIPH/VP;

[TABLE]
double DV = (CENT/VC) * (1 + EPS(1)) + EPS(2);
int i = 0;
while(DV<0 && i <100) {
simeps();
DV = (CENT/VC) * (1 + EPS(1)) + EPS(2);
++i;

}
```

```
[CAPTURE] DV CL VP VC Q
"

my_model <- mcode("mmf_model", code)
```

# Steady state dosing: 1000 mg every 12h for 22 doses
e1_1000_SS <- ev(ID = 1:1000, amt = 1000, ii = 12, ss = 1, addl = 21)

# Groups for 1000 mg
e1_1000 <- ev(ID = 1:1000, amt = 1000, ii = 12, ss = 1, addl = 1)
e2_1000 <- ev(ID = 1:1000, amt = 1000, ii = 12, addl = 0, ss = 0)
e3_1000 <- ev(ID = 1:1000, amt = 1000, ii = 12, addl = 9, ss = 0)

# Groups for 750 mg
e1_750 <- ev(ID = 1:1000, amt = 1000, ii = 12, ss = 1, addl = 1)
e2_750 <- ev(ID = 1:1000, amt = 1000, ii = 6, addl = 0, ss = 0)
e3_750 <- ev(ID = 1:1000, amt = 1000, ii = 12, addl = 9, ss = 0)

# Groups for 500 mg
e1_500 <- ev(ID = 1:1000, amt = 1000, ii = 12, ss = 1, addl = 1)
e2_500 <- ev(ID = 1:1000, amt = 1500, ii = 12, addl = 0, ss = 0)
e3_500 <- ev(ID = 1:1000, amt = 1000, ii = 12, addl = 9, ss = 0)

# Convert events to dataframes by time and ID
data_1000_SS <- as.data.frame(seq(e1_1000_SS)) %>% arrange(ID, time)
data_1000 <- as.data.frame(seq(e1_1000, wait=12, e2_1000, e3_1000)) %>% arrange(ID, time)
data_750 <- as.data.frame(seq(e1_750, wait=6, e2_750, e3_750)) %>% arrange(ID, time)
data_500 <- as.data.frame(seq(e1_500, wait=12, e2_500, e3_500)) %>% arrange(ID, time)

# Set seed for reproducibility
set.seed(191124)

# Simulations
sim_1000_SS <- my_model %>% data_set(data_1000_SS) %>% Req(DV) %>% mrgsim(delta = 0.5, end = 144)
sim_1000 <- my_model %>% data_set(data_1000) %>% Req(DV) %>% mrgsim(delta = 0.5, end = 144)
sim_750 <- my_model %>% data_set(data_750) %>% Req(DV) %>% mrgsim(delta = 0.5, end = 144)
sim_500 <- my_model %>% data_set(data_500) %>% Req(DV) %>% mrgsim(delta = 0.5, end = 144)

Convert results to tibble and assign group labels
```
```

```
sim_1000_df_ss <- as_tibble(sim_1000_SS) %>% mutate(group = "Steady State")
sim_1000_df <- as_tibble(sim_1000) %>% mutate(group = "Missed dose")
sim_750_df <- as_tibble(sim_750) %>% mutate(group = "Delay by 6h dose")
sim_500_df <- as_tibble(sim_500) %>% mutate(group = "Mitigation strategy")

# Combine all groups
combined_data <- bind_rows(sim_1000_df_ss, sim_1000_df, sim_750_df, sim_500_df)

# Redefine group levels in the desired order
combined_data$group <- factor(combined_data$group,
              levels = c("Steady State", "Missed dose", "Delay by 6h dose", "Mitigation
strategy"))

# Remove outliers
filtered_data <- combined_data %>%
 group_by(group) %>%
 mutate(
  p001 = quantile(DV, 0.01),
  p9999 = quantile(DV, 0.99)
 ) %>%
 ungroup() %>%
 dplyr::filter(DV >= p001 & DV <= p9999)

# Summary statistics by time and group
stats_PK_Rong <- filtered_data %>%
 group_by(time, group) %>%
 summarise(
  mean_DV = mean(DV),
  p5 = quantile(DV, 0.05),
  p95 = quantile(DV, 0.95),
  .groups = "drop"
 )

# Define colors for lines
custom_colors <- c(
 "Steady State" = "#000000",     # black
 "Missed dose" = "#E41A1C",      # bright red
 "Delay by 6h dose" = "#377EB8",  # medium blue
 "Mitigation strategy" = "#4DAF4A" # medium green
)

# Define fill colors for ribbons
custom_fills <- c(
 "Steady State" = "gray85",       # light gray
 "Missed dose" = "#FBB4AE",        # pastel red
 "Delay by 6h dose" = "#B3CDE3",    # pastel blue
 "Mitigation strategy" = "#CCEBC5"   # pastel green
```

```
)

# Define line types for mean curves
custom_linetypes <- c(
 "Steady State" = "dotted",
 "Missed dose" = "solid",
 "Delay by 6h dose" = "dashed",
 "Mitigation strategy" = "twodash"
)

# Create the plot
plot_PK_clean <- ggplot(stats_PK_Rong, aes(x = time)) +

 # P5–P95 ribbons without borders
 geom_ribbon(
  aes(ymin = p5, ymax = p95, fill = group),
  alpha = 0.4
 ) +

 # Mean curves with color and line types
 geom_line(
  aes(y = mean_DV, color = group, linetype = group),
  size = 1.2
 ) +

 # Custom palettes
 scale_color_manual(values = custom_colors) +
 scale_fill_manual(values = custom_fills) +
 scale_linetype_manual(values = custom_linetypes) +

 # Legends
 guides(
  color = guide_legend(title = "Scenarios"),
  linetype = guide_legend(title = "Scenarios"),
  fill = "none"
 ) +

 # Titles and axes
 labs(
  title = "MPA concentration over time across different scenarios",
  x = "Time (hours)",
  y = "MPA concentration (mg/L)"
 ) +

 # Theme
 theme_minimal(base_size = 10) +
 theme(
```

```
   legend.position = "bottom",
   legend.title = element_text(size = 10),
   legend.text = element_text(size = 9),
   axis.text = element_text(size = 9),
   axis.title = element_text(size = 10),
   plot.title = element_text(hjust = 0.5)
  ) +

  scale_x_continuous(breaks = seq(0, 144, by = 12))

# View the plot
plot_PK_clean

# Define the dosing events for each group

# 1250 mg group
e1_1000 <- ev(ID = 1:1000, amt = 1000, ii = 12, ss = 1, addl = 1)
e2_1000 <- ev(ID = 1:1000, amt = 1500, ii = 12, addl = 0, ss = 0)
e3_1000 <- ev(ID = 1:1000, amt = 1000, ii = 12, addl = 9, ss = 0)

# 1000 mg group
e1_750 <- ev(ID = 1:1000, amt = 750, ii = 12, ss = 1, addl = 1)
e2_750 <- ev(ID = 1:1000, amt = 1000, ii = 12, addl = 0, ss = 0)
e3_750 <- ev(ID = 1:1000, amt = 750, ii = 12, addl = 9, ss = 0)

# 750 mg group
e1_500 <- ev(ID = 1:1000, amt = 750, ii = 12, ss = 1, addl = 1)
e2_500 <- ev(ID = 1:1000, amt = 1250, ii = 12, addl = 0, ss = 0)
e3_500 <- ev(ID = 1:1000, amt = 750, ii = 12, addl = 9, ss = 0)

# Convert events to dataframes and sort by ID and time
data_1250_R <- as.data.frame(seq(e1_1000, wait = 12, e2_1000, e3_1000)) %>%
arrange(ID, time)
data_1000_R <- as.data.frame(seq(e1_750, wait = 12, e2_750, e3_750)) %>% arrange(ID,
time)
data_750_R <- as.data.frame(seq(e1_500, wait = 12, e2_500, e3_500)) %>% arrange(ID,
time)

# Set random seed for reproducibility
set.seed(260224)

# Run simulations
sim_1250_R <- my_model %>% data_set(data_1250_R) %>% Req(DV) %>%
mrgsim(delta = 1, end = 160)
sim_1000_R <- my_model %>% data_set(data_1000_R) %>% Req(DV) %>%
mrgsim(delta = 1, end = 160)
```

```
sim_750_R <- my_model %>% data_set(data_750_R) %>% Req(DV) %>% mrgsim(delta =
1, end = 160)

# Convert simulation results into tibbles
sim_1250_df_R <- as_tibble(sim_1250_R) %>% mutate(group = "1000 mg")
sim_1000_df_R <- as_tibble(sim_1000_R) %>% mutate(group = "750 mg")
sim_750_df_R <- as_tibble(sim_750_R) %>% mutate(group = "500 mg")

# Combine all simulation results into one dataframe
combined_sim_df_R <- bind_rows(sim_1250_df_R, sim_1000_df_R, sim_750_df_R)

# Function to calculate AUC by time interval
calcule_auc_par_intervalle <- function(data) {
 library(dplyr)
 library(purrr)
 library(stringr)

 cat("➡️ Calculating AUCs by interval...\n")

 results_forget <- data %>%
  arrange(ID, time) %>%
  group_by(ID, time) %>%
  slice_max(DV) %>%
  ungroup()

 start <- seq(0, 132, by = 12)
 end <- seq(12, 144, by = 12)

 auc_list <- map2(start, end, function(s, e) {
  results_forget %>%
   group_by(ID) %>%
   rename(id = ID) %>%
   makeAUC(DV ~ time, start = s, end = e) %>%
   mutate(interval = str_c(s, e, sep = "_"))
 })

 auc_result <- bind_rows(auc_list) %>%
  rename(auc = tau)  # Important: keep 'interval'

 return(auc_result)
}

# Function to identify return to steady state
identifier_retour_equilibre <- function(auc_df) {
 library(dplyr)
 library(stringr)
```

```
  cat("➡️ Identifying return to steady state (±10% around AUC 12–24 h)...\n")

  ref_auc <- auc_df %>%
    dplyr::filter(interval == "12_24") %>%
    select(id, auc_ref = auc)

  full_df <- auc_df %>%
    left_join(ref_auc, by = "id")

  retour_df <- full_df %>%
    mutate(
      lower_bound = auc_ref * 0.9,
      upper_bound = auc_ref * 1.1,
      within_range = auc >= lower_bound & auc <= upper_bound
    )

  retour_equilibre <- retour_df %>%
    dplyr::filter(within_range) %>%
    dplyr::filter(as.numeric(str_extract(interval, "^[0-9]+")) >= 24) %>%
    group_by(id) %>%
    slice_head(n = 1) %>%
    ungroup() %>%
    select(id, interval_return_to_steady_state = interval, auc, auc_ref)

  return(retour_equilibre)
}

# Apply the functions to each group
auc_par_intervalle_750 <- calcule_auc_par_intervalle(sim_750_df_R)
retour_equilibre_750 <- identifier_retour_equilibre(auc_par_intervalle_750)

auc_par_intervalle_1000 <- calcule_auc_par_intervalle(sim_1000_df_R)
retour_equilibre_1000 <- identifier_retour_equilibre(auc_par_intervalle_1000)

auc_par_intervalle_1250 <- calcule_auc_par_intervalle(sim_1250_df_R)
retour_equilibre_1250 <- identifier_retour_equilibre(auc_par_intervalle_1250)

# Define dosing events
e1 <- ev(ID = 1:1000, amt = 500, ii = 12, ss = 1, addl = 2)
e2 <- ev(ID = 1:1000, amt = 500, ii = 12, ss = 0, addl = 0)
e3 <- ev(ID = 1:1000, amt = 500, ii = 12, ss = 0, addl = 8)

# Define the sequence of events with a missed dose at the second interval
data_12H_750 <- seq(e1, wait = 12, e2, e3)

# Simulations
final_data <- as_tibble(data_12H_750) %>% arrange(ID)
```

```
set.seed(160424)

sim_forget <- my_model %>%
  data_set(final_data) %>%
  Req(DV) %>%
  mrgsim(delta = 1, end = 132)

# Keep the highest value at each timepoint for each subject
results_forget <- as_tibble(sim_forget) %>%
  arrange(ID, time) %>%
  group_by(ID, time) %>%
  slice_max(DV) %>%
  ungroup()

# AUC calculation per dosing interval
iterate_auc <- function(start, end) {
  results_forget %>%
    group_by(ID) %>%
    rename(id = ID) %>%
    makeAUC(DV ~ time, start = start, end = end) %>%
    mutate(tau = round(tau, 0))
}

start <- seq(0, 120, by = 12)
end <- seq(12, 132, by = 12)
names_iter <- str_c(start, end, sep = "_")

auc_res <- map2_df(start, end, iterate_auc) %>%
  rename(auc = tau) %>%
  arrange(id) %>%
  bind_cols(interval = rep(names_iter, 1000))

auc_res

# Calculate relative AUC changes from baseline
relative_auc_change <- auc_res %>%
  # Extract baseline AUC
  left_join(
    auc_res %>%
      group_by(id) %>%
      slice_head(n = 1) %>%
      select(auc_baseline = auc)
  ) %>%
  group_by(interval) %>%
  mutate(relative_auc = (auc - auc_baseline) / auc_baseline * 100) %>%
  select(-auc_baseline)
```

```
# Summary statistics for relative AUC change
mean_AUC <- relative_auc_change %>%
  summarise(
    mean_AUC = mean(relative_auc),
    sd_AUC = sd(relative_auc),
    minAUC = min(relative_auc),
    maxAUC = max(relative_auc),
    median_AUC = median(relative_auc)
  )

relative_auc_change

# Remove extreme outliers (1st and 99th percentiles)
quantiles <- quantile(auc_res$auc, probs = c(0.01, 0.99))

auc_result <- auc_res %>%
  dplyr::filter(auc > quantiles[1], auc < quantiles[2])

# Classify AUCs and compare between steady-state and missed dose
auc_final <- auc_result %>%
  left_join(
    auc_result %>%
      group_by(id) %>%
      slice(2) %>%
      select(id, auc_SS = auc)
  ) %>%
  group_by(id) %>%
  mutate(AUC_SS_group = cut(auc_SS, breaks = c(-Inf, 29.99, 60, Inf), labels = c("<30",
"30–60", ">60"))) %>%
  left_join(
    auc_result %>%
      group_by(id) %>%
      slice(4) %>%
      select(id, auc_missed = auc)
  ) %>%
  group_by(id) %>%
  mutate(AUC_missed_group = cut(auc_missed, breaks = c(-Inf, 29.99, 60, Inf), labels =
c("<30", "30–60", ">60")))

auc_final

# Identify balance loss after missed dose
data_frame <- auc_final %>%
  distinct(id, .keep_all = TRUE) %>%
  mutate(balance_loss = ifelse(
    auc_SS > 29.99 & auc_missed > 29.99,
    "AUC preserved (≥30 h·mg/L)",
```

```
    "AUC <30 h·mg/L"
  ))

# Remove missing values
data_clean <- na.omit(data_frame)

# Create histogram
graph_Normal_500_6 <- ggplot(data_clean, aes(x = balance_loss)) +
  geom_bar(color = "black", fill = "grey") +
  geom_text(stat = "count", aes(label = ..count..), vjust = -0.5) +
  labs(
    title = "750 mg twice daily: Distribution of equilibrium losses",
    x = "Loss of balance",
    y = "Number of cases"
  ) +
  theme_minimal() +
  theme(plot.title = element_text(hjust = 0.5))  # Center the title

graph_Normal_500_6
```

Population Pharmacokinetics of Mycophenolic Acid in Renal Transplant Recipients (Reinier, 2005)

```
code <-
"
[SET] end=100, delta=0.1

[PARAM] @annotated
TVTlag : 0.21 : Lag time (h)
TVKA : 4.1 : Typical absorption rate constant (1/h)
TVCL : 32.5 : Typical Clearance (L/h)
TVVC : 91 : Typical central volume (L)
TVQ : 35 : Intercompartmental clearance (L/h)
TVVP : 237 : Peripheral volume (L)
θCLCR : -0.62 : Creatinine clearance effect on VC
θALB : -1.13 : Albumin effect on VC
θCLcR : -0.12 : Creatinine clearance effect on CL
θGender : 1.11 : Gender covariate
θALBCL : -1.07 : Albumin effect on CL
θCIC : 0.31 : Ciclosporine dose effect on CL

[PARAM] @annotated @covariates
CLCR : 60 : Creatinine clearance (ml/min)
ALB : 40 : Plasma albumin (g/L)
CIC : 300 : Ciclosporine daily dose (mg)
Gender : 0 : Gender (0 = male, 1 = female)
```

```
[OMEGA] @annotated
ETAKA : 0.802 : IIV on Ka
ETACL : 0.091 : IIV on CL
ETAVC : 0.602 : IIV on VC
ETAVP : 0.712 : IIV on VP
ETA_KA : 0.86 : IPV on Ka
ETA_VC : 0.384 : IPV on VC
ETA_CL : 0.084 : IPV on CL

[SIGMA]
0.00000001
0.00000001

[CMT] @annotated
GUT : stomach [ADM]
CENT : Central compartment [OBS]
PERIPH : Peripheral compartment

[MAIN]
double CL = ((TVCL * pow(CLCR/48, θCLcR)) * pow(ALB/30, θALBCL) * pow(CIC/450,
θCIC) * pow(θGender, Gender)) * exp(ETACL + ETA_CL);
double VC = TVVC * pow(CLCR/48, θCLCR) * pow(ALB/30, θALB) * exp(ETAVC + ETA_VC);
double KA = TVKA * exp(ETAKA + ETA_KA);
double Q = TVQ;
double VP = TVVP * exp(ETAVP);
ALAG_GUT = TVTlag;

[ODE]
dxdt_GUT    = -KA * GUT;
dxdt_CENT   = -(CL + Q) * CENT / VC + Q * PERIPH / VP + KA * GUT;
dxdt_PERIPH = Q * CENT / VC - Q * PERIPH / VP;

[TABLE]
double DV = (CENT / VC) * (1 + EPS(1)) + EPS(2);
int i = 0;
while (DV < 0 && i < 100) {
  simeps();
  DV = (CENT / VC) * (1 + EPS(1)) + EPS(2);
  ++i;
}

[CAPTURE] DV CL VP VC Q
"

my_model_Reinier <- mcode("mmf_model_Reinier", code)

# Generate gender data for each subject
```

```r
set.seed(260224)
Gender_data <- tibble(ID = 1:1000, Gender = rbinom(1000, 1, 0.50))  # 0 = male, 1 =
female

# Define events for each dose group
# 1250 mg group
e1_1000 <- ev(ID = 1:1000, amt = 1250, ii = 12, ss = 1, addl = 1)
e2_1000 <- ev(ID = 1:1000, amt = 1250, ii = 12, addl = 0, ss = 0)
e3_1000 <- ev(ID = 1:1000, amt = 1250, ii = 12, addl = 9, ss = 0)

# 1000 mg group
e1_750 <- ev(ID = 1:1000, amt = 1000, ii = 12, ss = 1, addl = 1)
e2_750 <- ev(ID = 1:1000, amt = 1000, ii = 12, addl = 0, ss = 0)
e3_750 <- ev(ID = 1:1000, amt = 1000, ii = 12, addl = 9, ss = 0)

# 750 mg group
e1_500 <- ev(ID = 1:1000, amt = 750, ii = 12, ss = 1, addl = 1)
e2_500 <- ev(ID = 1:1000, amt = 750, ii = 12, addl = 0, ss = 0)
e3_500 <- ev(ID = 1:1000, amt = 750, ii = 12, addl = 9, ss = 0)

# Convert events to dataframes, sort and merge with gender
data_1250_R <- as.data.frame(seq(e1_1000)) %>% arrange(ID, time) %>%
left_join(Gender_data, by = "ID")
data_1000_R <- as.data.frame(seq(e1_750)) %>% arrange(ID, time) %>%
left_join(Gender_data, by = "ID")
data_750_R  <- as.data.frame(seq(e1_500)) %>% arrange(ID, time) %>%
left_join(Gender_data, by = "ID")

# Set seed for reproducibility
set.seed(260224)

# Simulations
sim_1250_R <- my_model_Reinier %>% data_set(data_1250_R) %>% Req(DV) %>%
mrgsim(delta = 0.1, end = 12)
sim_1000_R <- my_model_Reinier %>% data_set(data_1000_R) %>% Req(DV) %>%
mrgsim(delta = 0.1, end = 12)
sim_750_R  <- my_model_Reinier %>% data_set(data_750_R)  %>% Req(DV) %>%
mrgsim(delta = 0.1, end = 12)

# Convert simulation results to tibbles
sim_1250_df_R <- as_tibble(sim_1250_R) %>% mutate(group = "1250 mg")
sim_1000_df_R <- as_tibble(sim_1000_R) %>% mutate(group = "1000 mg")
sim_750_df_R  <- as_tibble(sim_750_R)  %>% mutate(group = "750 mg")

# Combine all simulations
combined_sim_df_Reinier <- bind_rows(sim_750_df_R, sim_1000_df_R, sim_1250_df_R)
```

```r
# Define group order
combined_sim_df_Reinier$group <- factor(combined_sim_df_Reinier$group,
                                        levels = c("750 mg", "1000 mg", "1250 mg"))

# Step 1: Remove extreme values (outside 1st and 99th percentiles) within each group
filtered_data <- combined_sim_df_Reinier %>%
  group_by(group) %>%
  mutate(
    p001 = quantile(DV, 0.01),
    p9999 = quantile(DV, 0.99)
  ) %>%
  ungroup() %>%
  dplyr::filter(DV >= p001 & DV <= p9999)

# Step 2: Summary statistics by time and group
stats_PK_Reinier <- filtered_data %>%
  group_by(time, group) %>%
  summarise(
    mean_DV = mean(DV),
    p5 = quantile(DV, 0.05),
    p95 = quantile(DV, 0.95),
    .groups = "drop"
  )

# Define grayscale color palette for clarity
gray_colors <- c("750 mg" = "gray10",   # nearly black
                 "1000 mg" = "gray40",  # medium gray
                 "1250 mg" = "gray70")  # lighter gray

# Plot
plot_PK_grayscale <- ggplot() +
  # Raw data points (very light)
  geom_point(data = filtered_data, aes(x = time, y = DV, color = group),
             alpha = 0.03, size = 0.5, show.legend = FALSE) +

  # 5th percentile line (thin dashed)
  geom_line(data = stats_PK_Reinier, aes(x = time, y = p5, color = group),
            linetype = "dashed", size = 0.6, show.legend = FALSE) +

  # 95th percentile line
  geom_line(data = stats_PK_Reinier, aes(x = time, y = p95, color = group),
            linetype = "dashed", size = 0.6, show.legend = FALSE) +

  # Mean line (thicker)
  geom_line(data = stats_PK_Reinier, aes(x = time, y = mean_DV, color = group),
            size = 1.2) +
```

```
 # Custom grayscale palette
 scale_color_manual(values = gray_colors) +

 # Legend
 guides(color = guide_legend(title = "Dosage group")) +

 # Axis labels
 labs(
  x = "Time (hours)",
  y = "MPA concentration (mg/L)"
 ) +

 # Minimal theme
 theme_minimal(base_size = 10) +
 theme(
  legend.position = "bottom",
  legend.title = element_text(size = 10),
  legend.text = element_text(size = 9),
  axis.text = element_text(size = 9),
  axis.title = element_text(size = 10),
  plot.title = element_text(hjust = 0.5)
 ) +

 # Time ticks every 2 hours
 scale_x_continuous(breaks = seq(0, 12, by = 2))

plot_PK_grayscale
```
