# Supplementary material for: Population pharmacokinetic modeling of missed mycophenolate mofetil doses: Impact on exposure and exploration of mitigation strategies
Source: PLoS One. 2025 Aug 28;20(8):e0330854. doi: 10.1371/journal.pone.0330854 (PMC12393703; doi:10.1371/journal.pone.0330854)
Supplement: Attachment — Submitted filename: Response to Reviewers_okMCA.docx [file pone.0330854.s003.docx]

**Response to Reviewers**

We thank the reviewers and the academic editor for their careful evaluation of our manuscript entitled:
**"Pharmacokinetic consequences and recovery strategies after missed or delayed doses of mycophenolate mofetil in kidney transplant recipients: a model-based simulation study."**
We are grateful for their constructive comments, which helped us improve our manuscript.
Below, we provide detailed responses to each of the comments raised, with references to the relevant lines in the revised manuscript.

**Reviewer #1**

This reviewer supported the publication of the manuscript in its current form. We thank them sincerely for their positive feedback and appreciation of the clinical relevance of our study.

**Reviewer #2**

**1/ Although the results are clearly presented, their practical implementation could be further supported. The key recommendations would benefit from being summarized in an accessible format, such as a decision table or clinical algorithm. A brief discussion of how these strategies could be integrated into patient education materials, discharge protocols, or therapeutic drug monitoring procedures would help reinforce their clinical utility.**

**Response:**
We agree with the reviewer that the clinical implementation of our findings is essential. We have added a clear reference to a new decision table (Table 4) summarizing the key model-informed recommendations. We also added a sentence explicitly discussing how these strategies could be integrated into clinical practice.
**Revised text (Line 302 of the clean version of the revised manuscript):**
*A summary table of these model-informed recommendations is provided below (Table 4). Integrating these recommendations into patient education resources, discharge procedures, or therapeutic drug monitoring practices could enhance their clinical applicability and support standardized responses to missed or delayed doses in routine care.*

| Clinical scenario | Recommended Action | Rationale |
| --- | --- | --- |
| Delay < 6 hours | Take the full dose as soon as the delay is identified | Has a minimal impact on the AUC; supports existing clinical recommendations |
| Delay > 6 hours  or  Fully missed dose | At the time of the intake following the delayed or missed dose: add half of the missed dose to the regular dose | Restores AUC _0-12h_ and limits the risk of underexposure |
| No half dose formulation available | Round down to the nearest available dose (e.g. 250 mg) | Reduces the risk of overexposure |

**2/ The authors fixed the residual error at a minimal value (σ = 0.001) to focus on inter-individual variability. While this is justifiable in the context of simulation, it likely underestimates the overall variability encountered in clinical settings, particularly among patients with fluctuating adherence or altered absorption. This limitation should be acknowledged explicitly, as it may affect the generalizability of the findings.**

**Response:**
We appreciate this important point and have clarified this limitation in the revised manuscript. We now explicitly state that fixing the residual error may underestimate real-life variability, especially in patients with altered adherence or absorption.
**Revised text (Line 339 of the clean version of the revised manuscript):**
*Secondly, in our simulations, we fixed the residual unexplained variability to a minimal value (σ = 0.001) to specifically focus on the consequences of inter-individual pharmacokinetic variability and adherence patterns. This may have led to an underestimation of the overall variability observed in clinical practice, especially in patients with fluctuating adherence or altered absorption.*

**3/ AUC values below the 0.1st and above the 99.9th percentiles were excluded, with reference to general recommendations on outlier handling. Although this is methodologically reasonable, the implications of this decision should be briefly discussed. Patients with extreme pharmacokinetic profiles such as those with low baseline exposure may be at greater risk of adverse consequences following a missed dose. Excluding these individuals could potentially limit the applicability of the findings to high-risk subgroups.**

**Response:**
We thank the reviewer for highlighting this. We have now included a discussion of the implications of excluding extreme AUC values, emphasizing that such profiles were rare (<0.2%), and that patients with low exposure remain a key target for TDM. We also highlight that the compensatory strategy was safe even in high-exposure profiles.
**Revised text (Line 343 of the clean version of the revised manuscript):**
*Thirdly, to reduce the influence of extreme simulations with unrealistic parameter combinations, AUC values below the 0.1^st^ and above the 99.9^th^ percentiles were excluded, following general methodological recommendations for outlier handling. Nonetheless, we acknowledge that this may slightly limit the generalizability of our results to patients at the extremes of pharmacokinetic variability. It is important to note, however, that such extreme profiles were rare in our simulations and represent only a very small fraction of the overall population (<0.2%). Importantly, individuals with low baseline exposure who tend to fall in the lower tail of the AUC distribution are also those most impacted by missed doses, with slower recovery and a greater risk of underexposure. These patients remain a key target population for therapeutic drug monitoring and enhanced adherence support. Conversely, individuals with very high baseline exposure (upper tail) are less vulnerable to the impact of a missed dose and are at lower risk of falling below the therapeutic window. Notably, our proposed compensatory strategy adding half the missed dose did not lead to simulated exposures exceeding toxic thresholds, even in high-exposure profiles. Therefore, while extreme profiles were not formally retained in the analysis, the simulations still capture the clinically relevant range of variability and support the robustness and safety of our recommendations across most patient scenarios.*

**4/ The study models isolated dosing deviations. In clinical practice, however, non-adherence is frequently recurrent or fluctuating. While such patterns are more complex to simulate, this should be noted as a limitation, and future work exploring the impact of repeated deviations could be proposed.**

**Response:**
We agree with the reviewer that recurrent or fluctuating non-adherence is highly relevant in real-world settings. We now explicitly acknowledge this limitation and propose it as an important direction for future research.
**Revised text (Line 362 of the clean version of the revised manuscript):**
*Finally, our study focused on isolated dosing deviations (i.e., single missed or delayed doses). In clinical practice, however, non-adherence is often recurrent, intermittent, or fluctuating over time particularly among certain subgroups such as adolescents, patients with limited health literacy, or those facing complex treatment regimens. Simulating such dynamic and individualized adherence patterns poses methodological challenges, especially in terms of variability propagation and long-term pharmacokinetic impact. Therefore, evaluating the cumulative consequences of repeated or clustered deviations, and the potential role of adaptive or personalized recovery strategies, could further enhance the clinical relevance and applicability of model-informed approaches in transplant medicine.*

We thank the reviewers again for their thoughtful comments, which have helped strengthen both the clinical relevance and methodological clarity of our manuscript. We hope that the revised version meets your expectations and remains suitable for publication in *PLOS ONE*.

Sincerely,
**Maizaud Franck on behalf of all authors**

Corresponding author:

Caroline Monchaud

Title: PharmD, PhD

ORCID: 0000-0002-9637-5813

Mail: caroline.monchaud@inserm.fr

Address:

CHU Limoges, Inserm U1248 P&T

Service de Pharmacologie, toxicologie et pharmacovigilance

Centre de Biologie et de Recherche en Santé

CHU de Limoges

2 avenue Martin Luther King

F-87042 Limoges cedex, France.

Phone : +33 5 55 05 61 40

Fax : +33 5 55 05 61 62
